# Retraction: Transition-metal-free synthesis of conjugated microporous polymers *via* amine-catalyzed Suzuki–Miyaura coupling reaction

**DOI:** 10.1039/d1sc90250d

**Published:** 2021-11-30

**Authors:** Qingmin Liu, Shangbin Jin, Bien Tan

**Affiliations:** MOE Key Laboratory of Material Chemistry for Energy Conversion and Storage, Hubei Key Laboratory of Material Chemistry and Service Failure, School of Chemistry and Chemical Engineering, Huazhong University of Science and Technology Wuhan 430074 China bien.tan@mail.hust.edu.cn; School of Chemical Engineering and Technology, Xi’an Jiaotong University Xianning West Road Xi’an Shaanxi 710049 China

## Abstract

Retraction of ‘Transition-metal-free synthesis of conjugated microporous polymers *via* amine-catalyzed Suzuki–Miyaura coupling reaction’ by Qingmin Liu *et al.*, *Chem. Sci.*, 2021, DOI: 10.1039/d1sc03970a.

We, the named authors, hereby wholly retract this *Chemical Science* article. This article reports the synthesis of conjugated microporous polymers using an amine-catalyzed Suzuki–Miyaura coupling reaction. This article builds upon findings first reported by Xu *et al.*^1^ and invokes the same mechanism for Pd-free polymer synthesis. Since the publication of our article in *Chemical Science*, we have been made aware of concerns regarding the conclusions of the above-mentioned *Nature Catalysis* article, disputing the mechanisms and claims that the reactions reported are not Pd-free.^2,3^ We, as the authors of this *Chemical Science* article, have considered these claims and, after further investigation, acknowledge that we cannot exclude the influence of residual Pd for the Suzuki coupling reaction involved. Therefore, there is sufficient doubt around the main conclusion of our work as a ‘transition-metal-free synthesis’ and so we wish to retract this article.

Signed: Qingmin Liu, Shangbin Jin and Bien Tan, 18th November 2021.

Retraction endorsed by May Copsey, Executive Editor, *Chemical Science*.

## Supplementary Material
